# A stent too far: endoscopic ultrasonography-guided duodenojejunostomy for malignant duodenal obstruction after placement of a stent in the horizontal part

**DOI:** 10.1055/a-2512-5082

**Published:** 2025-02-05

**Authors:** Zheng Zhao, Zheng Zhang, Xinghua Zhang, Anni Zhou, Peng Li

**Affiliations:** 1 Department of Gastroenterology, Beijing Friendship Hospital, Capital Medical University; State Key Laboratory for Digestive Health; National Clinical Research Center for Digestive Diseases, Beijing, China

Endoscopic ultrasonography-guided gastroenterostomy offers distinctive benefits in the management of malignant gastric outlet obstruction 11 22 . When faced with unusual obstruction, the endoscopist must find an appropriate place to “build a bridge” 33 . Here, we report a case of EUS-guided duodenojejunostomy following placement of a duodenal stent in the horizontal part.

A 60-year-old man with a history of pancreatic cancer was admitted because of long-term indigestion and acute melena. The patient had received chemotherapy and endoscopic retrograde biliary drainage for jaundice 2 years previously, and duodenal stent placement (at the horizontal part) for malignant obstruction 1 year ago. Esophagogastroduodenoscopy and computed tomography showed severe tumor ingrowth ( Fig. 1Fig. 1 **a** , Fig. 2Fig. 2 ) and active bleeding at the distal end of the stent ( Fig. 1Fig. 1 **b** ).

**Fig. 1:**
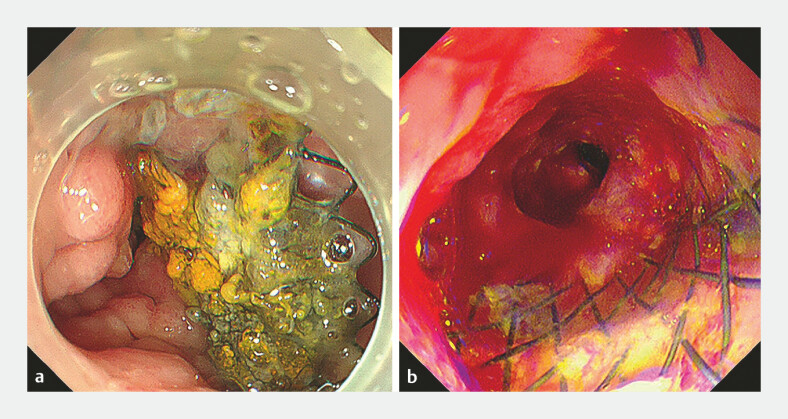
**Fig. 1** Endoscopic images showing tumor ingrowth and bleeding. **a** The inner lumen of the duodenal stent was narrowed by tumor ingrowth. **b** An active oozing hemorrhage was found at the distal end of the stent.

**Fig. 2:**
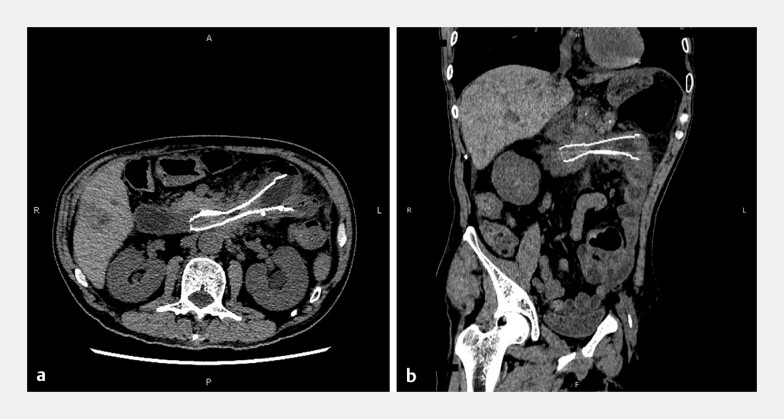
**Fig. 2** Abdominal computed tomography image of the narrowed duodenal stent. **a** Axial view. **b** Reconstruction image.

Given the poor general status and the duodenal obstruction, a double-balloon-assisted gastrojejunostomy was planned after complete hemostasis. Unexpectedly, the distance between the stomach and the jejunum was too great for anastomosis, and the operation approach was blocked by the stent ( Fig. 3Fig. 3 **a** ). After repeated attempts, the distance between the duodenal bulb and the distal balloon was close enough to place the stent ( Fig. 3Fig. 3 **b** ). EUS-guided puncture of the distal balloon was performed transduodenally with the Hot Axios system (M00553550; Boston Scientific, Marlborough, Massachusetts, USA), and the stent was then delivered into the jejunum and deployed over the guidewire. A guidewire was then successfully advanced through the stent under fluoroscopy. Finally, with the injection of contrast agent, a clear delineation of the jejunum confirmed the duodenojejunostomy ( Fig. 4Fig. 4 , Video 1Video 1 ). Postoperatively, the patient presented relief of obstructive symptoms and good stent patency by barium radiography ( Fig. 5Fig. 5 ).

**Fig. 3:**
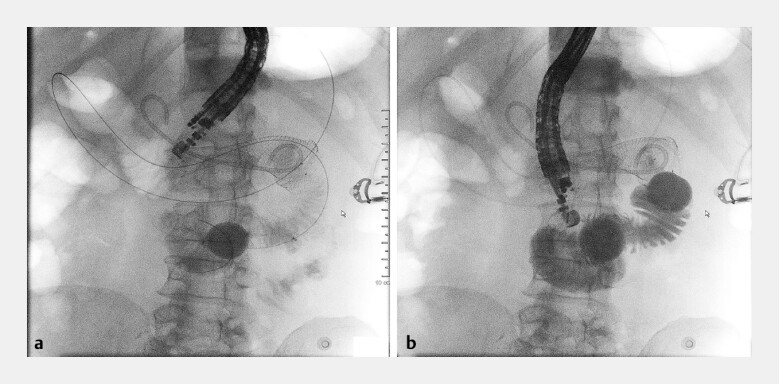
**Fig. 3** Procedure for identifying the site of puncture. **a** The distance between the stomach and the jejunum was too great for anastomosis. **b** The distance between the duodenal bulb and the distal balloon was appropriate for stent placement.

**Fig. 4:**
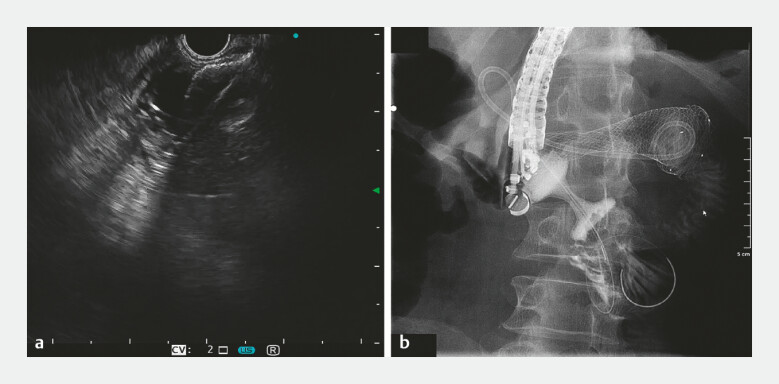
**Fig. 4** Procedure for endoscopic ultrasonography-guided duodenojejunostomy. **a** Echoendoscopic view of stent deployment. **b** A guidewire was advanced into the jejunum through the stent.

**Fig. 5:**
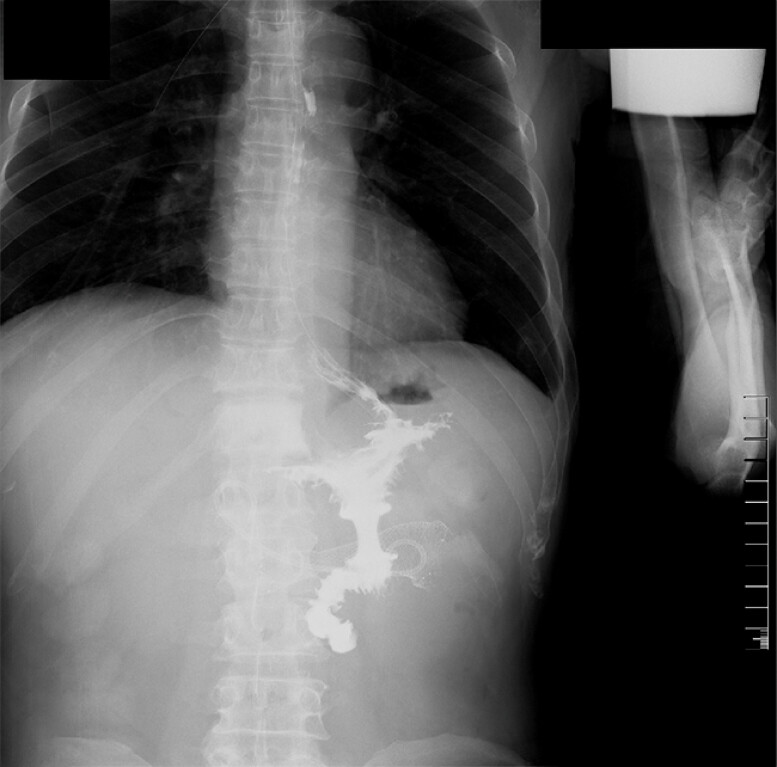
**Fig. 5** Postoperative barium radiography image.

Endoscopic ultrasonography-guided duodenojejunostomy for malignant duodenal obstruction after placement of a stent in the horizontal part.Video 1Video 1

The field of therapeutic EUS has expanded greatly 44 . In this case, we highlight a particular EUS-guided duodenojejunostomy procedure demonstrating the endoscopist’s creativity. Sometimes, the endoscopist must become a pathfinder to deal with the complex conditions in the gastrointestinal tract.

Endoscopy_UCTN_Code_TTT_1AS_2AB

## Notes

Endoscopy E-Videos https://eref.thieme.de/e-videos
*E-Videos* is an open access online section of the journal *Endoscopy* , reporting on interesting cases and new techniques in gastroenterological endoscopy. All papers include a high-quality video and are published with a Creative Commons CC-BY license. Endoscopy E-Videos qualify for HINARI discounts and waivers and eligibility is automatically checked during the submission process. We grant 100% waivers to articles whose corresponding authors are based in Group A countries and 50% waivers to those who are based in Group B countries as classified by Research4Life (see: https://www.research4life.org/access/eligibility/ ). This section has its own submission website at https://mc.manuscriptcentral.com/e-videos .

## References

[1] Chan SM Dhir V Chan YYY Endoscopic ultrasound-guided balloon-occluded gastrojejunostomy bypass, duodenal stent or laparoscopic gastrojejunostomy for unresectable malignant gastric outlet obstruction Dig Endosc 2023 35 512 519 10.1111/den.14472 36374127

[2] Teoh AYB Lakhtakia S Tarantino I Endoscopic ultrasonography-guided gastroenterostomy versus uncovered duodenal metal stenting for unresectable malignant gastric outlet obstruction (DRA-GOO): a multicentre randomised controlled trial Lancet Gastroenterol Hepatol 2024 9 124 132 10.1016/S2468-1253(23)00242-X 40347959

[3] Hanscom M Schmidt A Cherng N EUS-guided duodenojejunostomy for the nonsurgical management of duodenal obstruction in a patient with complicated postsurgical anatomy Gastrointest Endosc 2021 94 1010 1011 10.1016/j.gie.2021.07.015 34310922

[4] van der Merwe SW van Wanrooij RLJ Bronswijk M Therapeutic endoscopic ultrasound: European Society of Gastrointestinal Endoscopy (ESGE) Guideline Endoscopy 2022 54 185 205 10.1055/a-1717-1391 34937098

